# Acoustic wave and eikonal equations in a transformed metric space for various types of anisotropy

**DOI:** 10.1016/j.heliyon.2017.e00260

**Published:** 2017-03-22

**Authors:** Marcus M. Noack, Stuart Clark

**Affiliations:** a Kalkulo AS, Simula Research Laboratory, P.O.Box 134, 1325 Lysaker, Norway; b Department of Informatics, University of Oslo, Gaustadalleen 23 B, 0373 Oslo, Norway

**Keywords:** Geophysics, Acoustics

## Abstract

Acoustic waves propagating in anisotropic media are important for various applications. Even though these wave phenomena do not generally occur in nature, they can be used to approximate wave motion in various physical settings. We propose a method to derive wave equations for anisotropic wave propagation by adjusting the dispersion relation according to a selected type of anisotropy and transforming it into another metric space. The proposed method allows for the derivation of acoustic wave and eikonal equations for various types of anisotropy, and generalizes anisotropy by interpreting it as a change of the metric instead of a change of velocity with direction. The presented method reduces the scope of acoustic anisotropy to a selection of a velocity or slowness surface and a tensor that describes the transformation into a new metric space. Experiments are shown for spatially dependent ellipsoidal anisotropy in homogeneous and inhomogeneous media and sandstone, which shows vertical transverse isotropy. The results demonstrate the stability and simplicity of the solution process for certain types of anisotropy and the equivalency of the solutions.

## 1 Introduction

Anisotropic wave propagation has a variety of different applications because, compared with isotropic wave propagation, it is a more general representation of wave propagation and is valid in a wider range of materials. For instance, recent scientific attention has been focused on the development of devices that can appear to cloak objects. Prototypes of such devices have been constructed that bend certain wavelengths of light using metamaterials [1], [2]. In the case of acoustic waves, a device for preferential propagation of the sound through one of two possible and symmetrically aligned channels has been constructed, leading to effective anisotropic propagation of the wave [3]. Other metamaterials enhance the amplitude of sound waves before entering a microphone to enhance sensors [4]. These metamaterials are composed of sub-wavelength meta-atoms that lead to wavelength-scale effects such as non-reciprocal propagation of waves, negative refraction and wave isolation [5], [6]. Current cloaking technology is based on the concept of bending the wave around an object such that properties of the wave do not change, rendering the object in between the source and the sensor undetectable. As such, coordinate transformations that maintained the invariance of the wave properties have been defined, for electromagnetic waves [7], elasto-dynamic waves [8] and for acoustic waves in 2D [9] and 3D [10]. In addition, there exists a mathematical similarity between acoustic waves (longitudinal, compressional or P-waves) and transverse or s-waves through mapping the material properties [11]. Therefore, the modeling of anisotropic acoustic waves can be used to study several types of wave propagation. In this paper, we present a new and generic approach to model anisotropic acoustic wave propagation based on metric space transformations.

Metric space transformations have been proposed to handle anisotropy before. Dellinger [12] proposed to stretch a circle to model elliptical anisotropy, while Joets and Ribotta [13] applied the idea for the propagation of light in anisotropic media where the ray trajectories are the geodesics of an anisotropic metric space. Borovskikh [14] used the duality of anisotropy and metric space for a theoretical treatment of various eikonal equations for anisotropic media.

The first advancements in anisotropic wave propagation were made by physicists in the 19th century to investigate the propagation of light; however the major advances in anisotropic wave propagation were in the field of seismology [15]. The full anisotropic behavior is defined by a fourth-order tensor $c_{ijkl}$ to relate stress and strain. Due to the inherent complexity of this tensor, Voigt [16] noticed symmetries that allowed the 3×3×3×3 tensor to be reduced to a 6×6 symmetric matrix $C_{\alpha \beta }$. For particular anisotropic materials, the number of potential coefficients is further reduced; to 5, for example, for transversely isotropic materials, or to 9 for orthorhombic media [17]. For three-dimensional metric space transformations, 9 coefficients must be defined, as we will show below. In the next section, a method is presented that generalizes the procedure of deriving wave and eikonal equations for different kinds of anisotropy.

In nature, acoustic media does not physically admit body waves with anisotropic behavior; only a wave traveling along a curved manifold or through a moving medium can exhibit anisotropic behavior. However, it is possible to construct wave and eikonal equations for acoustic, anisotropic wave propagation by using the dispersion relation of the wave equation [18], [19]. The idea is to design a metric space for which the media properties are isotropic [14]. Hence, using the duality of anisotropic media and metric space, the anisotropic case can be treated like the isotropic one. Therefore, the benefits of the solution of the acoustic wave equation in isotropic media, like the simplicity of the mathematical treatment and the stability of the numerical solution, are inherited by the resulting equations for anisotropy. For elastic media, the acoustic wave equation is used as an approximation of the plane wave (P-wave) motion [18], while ignoring shear waves [19], [18]. Other situations in which anisotropic, acoustic wave propagation can be encountered are electric waves in muscle tissue [20], [21] and acoustic waves in moving media.

A metric space is in general defined by a set for which distances between all elements are defined. The definition of the distance between elements of the set is called a metric. The metric induces a topology on the set which leads to our definition of anisotropy. The duality of a metric space and anisotropy shall in this paper be used to generalize various types of anisotropy into one theory. The proposed unified theory can be used to derive simple, stable and efficient numerical solvers for wave and eikonal equations, and for a better theoretical understanding of anisotropy. A form of stretching the elliptical anisotropy iteratively to obtain a solution to the transversely isotropic eikonal equation can be found in Waheed et al. [22].

**Figure 1:**
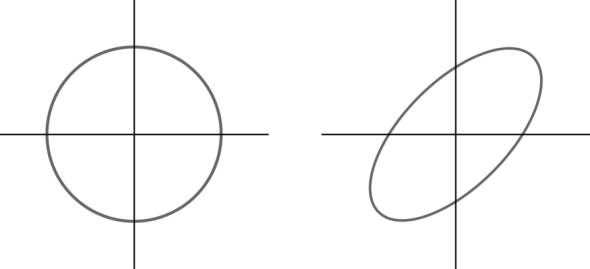
A comparison of a circle in different metric spaces. The circle on both sides of the figure is the set of all points satisfying *x*^2^ + *y*^2^ = 1. The underlying metric on the left side is the euclidean metric. The underlying metric on the right side is obtained by compressing and rotating the corresponding basis by 45 degrees with respect to the standard basis.

In homogeneous media, a sphere describes the velocity surface for the isotropic case. For ellipsoidal anisotropy, the sphere can directly be transformed into the new metric space by using the corresponding basis (Figure 1). Another version of this transformation was also used in Stovas and Alkhalifah [23]. This principle can be adapted for other types of anisotropy by using different $L^{p}$ norms for the computation of distances. At first, a surface must be chosen to describe the wave front, which can then be stretched and tilted by transforming it into another basis for the velocity. From the resulting surface, a dispersion relation can be derived which leads to the corresponding wave equation. From the wave equation, the corresponding eikonal equation can be obtained. The eikonal approximation provides information about first arrivals [24], does not account for caustics [25] and requires a sufficiently well defined source [26] if amplitudes are of interest. Even so, eikonal models are widely used in many fields as approximation due to their simplicity [27], [28]. Additional to the approximation of wave propagation, the solution of the eikonal equation has many other applications in a large variety of fields [29], [26], [30], [14], [31], [32], [33], [21].

The proposed theory can be derived by using a new basis for the slowness or the velocity; however, the chosen basis for the velocity in this work is more illustrative. The proposed method generalizes various types of anisotropy and offers a simple derivation, implementation and application since the given tensor field at each model point is illustrative, and dealing with angles between semi-principal axes and coordinate system axes [19] can be avoided. There is a natural limitation of the method due to the definition of a metric space. All elements of the set have to have a well (uniquely) defined distance between them. Therefore, triplications can not be accounted for. However, triplications only occur in very rare cases under certain circumstances and are therefore seldom considered in practice [34].

The remainder of the paper is organized as follows. Firstly, the theory section gives an overview of the idea and the physical background. Starting with the dispersion relation of the acoustic wave equation, a new wave equation for tilted ellipsoidal anisotropy is derived and generalized for other types of anisotropy. The results section shows five examples to illustrate the functionality of the method, including solutions for homogeneous and inhomogeneous anisotropic velocity fields and field specific examples.

## 2 Theory

**Figure 2:**
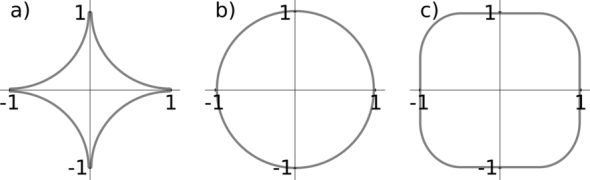
The figure shows different shapes of super-ellipses in the form 1 = *x*^*n*^ + *y*^*m*^. Super-ellipses can be used to describe slowness or velocity surfaces in the phase space with coordinates *p*_1_, *p*_2_, *p*_3_ or *v*_1_, *v*_2_, *v*_3_ respectively. Shape of the super-ellipse with a) $n=m=\frac{1}{2}$, b) *n* = *m* = 2, c) *n* = *m* = 4. Note that for non-integer exponents the procedure would lead to fractional derivatives in the wave equation which are numerically more difficult to handle. Super-ellipse surfaces are shown here as simple examples for possible velocity or slowness surfaces. However, the method is applicable to various surface shapes.

The theoretical treatment starts with the dispersion relation

(1) $$\omega^{2}=k_{1}^{2}+k_{2}^{2}+k_{3}^{2},$$

of the acoustic wave equation in three dimensions

(2) $$\frac{\partial^{2}u(x,t)}{\partial t^{2}}=c^{2}\nabla^{2}u(x,t),$$

where *ω* is the angular frequency, *c* is the wave velocity, $k_{i}$ is the wave number in the direction *i*, $\nabla^{2}$ is the Laplacian operator and u(x,t) is a scalar function. Equation (1) can be divided by $\omega^{2}$ and represents a slowness surface

(3) $$1=|p_{1}{|}^{2}+|p_{2}{|}^{2}+|p_{3}{|}^{2},$$

where $p_{i}=k_{i}/\omega$. The slowness surface in the form (3) represents a spherical wave front in the phase space with coordinates $p_{1},\;p_{2},\;p_{3}$ of an acoustic wave, traveling in homogeneous media with the wave velocity v=1 m/s for a travel time of T=1 s [35]. From this idea, various slowness surfaces can be constructed depending on the anisotropy one wants to model. The surfaces are in general not restricted to sixth-order polynomials like surfaces for waves in an elastic medium. Even though the method can be applied to a large number of surfaces, the focus in this work will be on velocity surfaces that can be described as a super-ellipsoid (Figure 2) in the form

(4) $$1={\left|\frac{x_{1}}{a}\right|}^{n}+{\left|\frac{x_{2}}{b}\right|}^{n}+{\left|\frac{x_{3}}{c}\right|}^{n},$$

since the resulting derivation of the corresponding wave equations are mathematically simpler and the numerical treatment is less complicated. In Equation (4), *a*,*b* and *c* represent the lengths of the semi-principal axes, which equal one in our case since the stretching is performed by the transformation into a new metric space. For n=2 equation (4) represents a sphere and will build the basis for tilted ellipsoidal anisotropy. From the slowness surface the corresponding dispersion relation can be derived.

The procedure to derive an acoustic wave equation in anisotropic media will be described using the example of tilted ellipsoidal anisotropy. The starting point is the dispersion relation for an isotropic medium (1) which must be transformed into a new metric space for the velocity. The corresponding tensor describing a new basis is given as

(5) $$\overset{ˆ}{V}(x)=\left(\begin{array}{ccc} {\overset{ˆ}{V}}_{11}(x) & {\overset{ˆ}{V}}_{12}(x) & {\overset{ˆ}{V}}_{13}(x)\\ {\overset{ˆ}{V}}_{21}(x) & {\overset{ˆ}{V}}_{22}(x) & {\overset{ˆ}{V}}_{23}(x)\\ {\overset{ˆ}{V}}_{31}(x) & {\overset{ˆ}{V}}_{32}(x) & {\overset{ˆ}{V}}_{33}(x) \end{array}\right),$$

where ${\overset{ˆ}{V}}_{i1},\;{\overset{ˆ}{V}}_{i2},\;{\overset{ˆ}{V}}_{i3}$ are orthogonal vectors of the new basis. The tensor $\overset{ˆ}{V}$ describes a possibly spatially dependent basis and leads to a new group velocity surface at each point in space, like the standard basis for the Euclidean space gives the group (and phase) velocity surface for isotropic wave propagation; therefore, it will be referred to as velocity tensor in the course of the paper. The velocity tensor defines a metric space at each model point. The corresponding metric space shall be called the velocity space. The velocity tensor must not be confused with the velocity itself which can be set to 1 m/s everywhere. The dispersion relation (1) can also be transformed to a new basis $\overset{ˆ}{S}$ by applying

(6) $${\overset{ˆ}{S}}^{-1}k=\left(\begin{array}{c} {\overset{ˆ}{S}}_{11}^{-1}k_{1}+{\overset{ˆ}{S}}_{12}^{-1}k_{2}+{\overset{ˆ}{S}}_{13}^{-1}k_{3}\\ {\overset{ˆ}{S}}_{21}^{-1}k_{1}+{\overset{ˆ}{S}}_{22}^{-1}k_{2}+{\overset{ˆ}{S}}_{23}^{-1}k_{3}\\ {\overset{ˆ}{S}}_{31}^{-1}k_{1}+{\overset{ˆ}{S}}_{32}^{-1}k_{2}+{\overset{ˆ}{S}}_{33}^{-1}k_{3} \end{array}\right),$$

where $\overset{ˆ}{S}$ is a tensor for the new basis that yields the slowness surface and will be referred to as slowness tensor in the course of the paper. The slowness tensor describes a corresponding metric space that shall be called the slowness space. The translation of the velocity space into the slowness space can, for ellipsoidal anisotropy, be approximated by preserving the direction of each basis vector and inverting its length and is given by

(7) $${\overset{ˆ}{S}}_{ij}=\frac{{\overset{ˆ}{V}}_{ij}}{{\overset{ˆ}{V}}_{1j}^{2}+{\overset{ˆ}{V}}_{2j}^{2}+{\overset{ˆ}{V}}_{3j}^{2}}.$$

The slowness space can tilt and stretch the slowness surface just like the velocity space $\overset{ˆ}{V}$ can stretch and tilt the velocity surface. The components of the vector of equation (6) are the $k_{i}$s in the new metric space. Therefore, inserting the vector components (6) in the dispersion relation (1) yields

(8) $$\begin{array}{cc} \omega^{2}= & {\left({\overset{ˆ}{S}}_{11}^{-1}k_{1}+{\overset{ˆ}{S}}_{12}^{-1}k_{2}+{\overset{ˆ}{S}}_{13}^{-1}k_{3}\right)}^{2}+\\ & {\left({\overset{ˆ}{S}}_{21}^{-1}k_{1}+{\overset{ˆ}{S}}_{22}^{-1}k_{2}+{\overset{ˆ}{S}}_{23}^{-1}k_{3}\right)}^{2}+\\ & {\left({\overset{ˆ}{S}}_{31}^{-1}k_{1}+{\overset{ˆ}{S}}_{32}^{-1}k_{2}+{\overset{ˆ}{S}}_{33}^{-1}k_{3}\right)}^{2}. \end{array}$$

Multiplying both sides of equation (8) with the wave field in the Fourier domain u(k,ω) and performing an inverse Fourier transformation ($k_{i}\to -j\frac{\partial }{\partial x_{i}},\;\omega \to j\frac{\partial }{\partial t}$), where $j=\sqrt{-1}$, leads to the acoustic wave equation for tilted ellipsoidal anisotropy

(9) $$\begin{array}{cc} \frac{\partial^{2}u(x,t)}{\partial t^{2}}= & \\ & \frac{\partial^{2}u(x,t)}{\partial x_{1}^{2}}({\overset{ˆ}{S}}_{11}^{-1}+{\overset{ˆ}{S}}_{21}^{-1}+{\overset{ˆ}{S}}_{31}^{-1})+\\ & \frac{\partial^{2}u(x,t)}{\partial x_{2}^{2}}({\overset{ˆ}{S}}_{12}^{-1}+{\overset{ˆ}{S}}_{22}^{-1}+{\overset{ˆ}{S}}_{32}^{-1})+\\ & \frac{\partial^{2}u(x,t)}{\partial x_{3}^{2}}({\overset{ˆ}{S}}_{13}^{-1}+{\overset{ˆ}{S}}_{23}^{-1}+{\overset{ˆ}{S}}_{33}^{-1})+\\ & 2\frac{\partial^{2}u(x,t)}{\partial x_{1}\partial x_{2}}({\overset{ˆ}{S}}_{11}^{-1}{\overset{ˆ}{S}}_{12}^{-1}+{\overset{ˆ}{S}}_{21}^{-1}{\overset{ˆ}{S}}_{22}^{-1}+{\overset{ˆ}{S}}_{31}^{-1}{\overset{ˆ}{S}}_{32}^{-1})+\\ & 2\frac{\partial^{2}u(x,t)}{\partial x_{1}\partial x_{3}}({\overset{ˆ}{S}}_{11}^{-1}{\overset{ˆ}{S}}_{13}^{-1}+{\overset{ˆ}{S}}_{21}^{-1}{\overset{ˆ}{S}}_{23}^{-1}+{\overset{ˆ}{S}}_{31}^{-1}{\overset{ˆ}{S}}_{33}^{-1})+\\ & 2\frac{\partial^{2}u(x,t)}{\partial x_{2}\partial x_{3}}({\overset{ˆ}{S}}_{12}^{-1}{\overset{ˆ}{S}}_{13}^{-1}+{\overset{ˆ}{S}}_{22}^{-1}{\overset{ˆ}{S}}_{23}^{-1}+{\overset{ˆ}{S}}_{32}^{-1}{\overset{ˆ}{S}}_{33}^{-1}). \end{array}$$

Since this derivation leads to a wave equation that is only valid at one model point **x**, $\overset{ˆ}{S}$ in equation (8) can be treated as a spatial constant. Equation (9) represents the acoustic wave equation for tilted ellipsoidal anisotropy. It can also be seen as an acoustic wave equation describing a wave traveling in isotropic media in a given metric space. The two formulations illustrate the duality of anisotropy and metric spaces. The presented procedure is similar for any other chosen velocity or slowness surface and therefore for many types of anisotropy.

For illustrative reasons, an alternative approach is shown to derive equation (9). The same result can be obtained by using the acoustic wave equation (2) directly and transforming the differential operator ($\partial /\partial x_{i}$) into the slowness space. This approach leads to

(10) $${\overset{ˆ}{S}}^{-1}\left(\begin{array}{c} \frac{\partial }{\partial x}\\ \frac{\partial }{\partial y}\\ \frac{\partial }{\partial z} \end{array}\right)=\left(\begin{array}{c} {\overset{ˆ}{S}}_{11}^{-1}\frac{\partial }{\partial x}+{\overset{ˆ}{S}}_{12}^{-1}\frac{\partial }{\partial y}+{\overset{ˆ}{S}}_{13}^{-1}\frac{\partial }{\partial z}\\ {\overset{ˆ}{S}}_{21}^{-1}\frac{\partial }{\partial x}+{\overset{ˆ}{S}}_{22}^{-1}\frac{\partial }{\partial y}+{\overset{ˆ}{S}}_{23}^{-1}\frac{\partial }{\partial z}\\ {\overset{ˆ}{S}}_{31}^{-1}\frac{\partial }{\partial x}+{\overset{ˆ}{S}}_{32}^{-1}\frac{\partial }{\partial y}+{\overset{ˆ}{S}}_{33}^{-1}\frac{\partial }{\partial z} \end{array}\right).$$

Inserting the new differential operators (10) in the acoustic wave equation (2) leads to the same result as equation (9). This equivalent approach shows that the only difference between the isotropic and the tilted ellipsoidal anisotropic case is the underlying metric space.

From equation (9) the eikonal equation

(11) $$\begin{array}{cc} 1= & {\left(\frac{\partial T(x)}{\partial x_{1}}\right)}^{2}({\overset{ˆ}{S}}_{11}^{-1}+{\overset{ˆ}{S}}_{21}^{-1}+{\overset{ˆ}{S}}_{31}^{-1})+\\ & {\left(\frac{\partial T(x)}{\partial x_{2}}\right)}^{2}({\overset{ˆ}{S}}_{12}^{-1}+{\overset{ˆ}{S}}_{22}^{-1}+{\overset{ˆ}{S}}_{32}^{-1})+\\ & {\left(\frac{\partial T(x)}{\partial x_{1}}\right)}^{2}({\overset{ˆ}{S}}_{13}^{-1}+{\overset{ˆ}{S}}_{23}^{-1}+{\overset{ˆ}{S}}_{33}^{-1})+\\ & 2\frac{\partial T(x)}{\partial x_{1}}\frac{\partial T(x)}{\partial x_{2}}({\overset{ˆ}{S}}_{11}^{-1}{\overset{ˆ}{S}}_{12}^{-1}+{\overset{ˆ}{S}}_{21}^{-1}{\overset{ˆ}{S}}_{22}^{-1}+{\overset{ˆ}{S}}_{31}^{-1}{\overset{ˆ}{S}}_{32}^{-1})+\\ & 2\frac{\partial T(x)}{\partial x_{1}}\frac{\partial T(x)}{\partial x_{3}}({\overset{ˆ}{S}}_{11}^{-1}{\overset{ˆ}{S}}_{13}^{-1}+{\overset{ˆ}{S}}_{21}^{-1}{\overset{ˆ}{S}}_{23}^{-1}+{\overset{ˆ}{S}}_{31}^{-1}{\overset{ˆ}{S}}_{33}^{-1})+\\ & 2\frac{\partial T(x)}{\partial x_{2}}\frac{\partial T(x)}{\partial x_{3}}({\overset{ˆ}{S}}_{12}^{-1}{\overset{ˆ}{S}}_{13}^{-1}+{\overset{ˆ}{S}}_{22}^{-1}{\overset{ˆ}{S}}_{23}^{-1}+{\overset{ˆ}{S}}_{32}^{-1}{\overset{ˆ}{S}}_{33}^{-1}) \end{array}$$

can be derived. Equation (11) can be used to compute the travel times of a wave front propagating in media with tilted ellipsoidal anisotropy.

The translation of a velocity surface into the corresponding slowness surface is a non-trivial problem since the actual slowness surface is created by inverting the radii in all directions and can no longer be described by taking the polynomial surface for the velocity and transforming it into the slowness space. For better understanding, we can have a look at ellipsoidal anisotropy. In the case of ellipsoidal anisotropy, the ellipsoid with semi-principal axes a, b, c describing the velocity surface leads to an ellipsoid with semi-principal axes 1/a, 1/b, 1/c describing the slowness surface even though this is only correct along the axes. It is in general not the case that the actual slowness surface which is obtained by inverting the radii of the velocity surface in all directions, resembles the slowness surface that is obtained by inverting the length of the axes. This approximation was used to preserve the simplicity of the method and leads to inaccuracies in the space between the axes. The issue seems less problematic if the velocity and slowness surfaces are considered to be approximations in practice and the real surfaces are unknown. Therefore, in the case of ellipsoidal anisotropy, the errors made by inverting only the radii in axes direction is smaller than the error made by approximating anisotropy as a known surface. For other surfaces the induced error can be larger. Another way to work around the problem is to give the slowness surface and the slowness tensor in the first step of the solution process, thereby omitting the need to translate between velocity and slowness. In this work, the velocity is chosen as a starting point for illustrative reasons. The issue of translating between velocity and slowness surfaces will be addressed in a more descriptive way in the next sections.

Using the procedure described above, a wave equation can be given for any super-ellipsoidal slowness surface

(12) $$\begin{array}{cc} \frac{\partial^{\phi }u(x,t)}{\partial t^{\phi }}=F^{-1}[( & |{\overset{ˆ}{S}}_{11}^{-1}k_{1}+{\overset{ˆ}{S}}_{12}^{-1}k_{2}+{\overset{ˆ}{S}}_{13}^{-1}k_{3}{|}^{\phi }\\ + & |{\overset{ˆ}{S}}_{21}^{-1}k_{1}+{\overset{ˆ}{S}}_{22}^{-1}k_{2}+{\overset{ˆ}{S}}_{23}^{-1}k_{3}{|}^{\phi }\\ + & |{\overset{ˆ}{S}}_{31}^{-1}k_{1}+{\overset{ˆ}{S}}_{32}^{-1}k_{2}+{\overset{ˆ}{S}}_{33}^{-1}k_{3}{|}^{\phi })]u(x,t), \end{array}$$

with the associated eikonal equation

(13) $$\begin{array}{cc} 1=( & |{\overset{ˆ}{S}}_{11}^{-1}\frac{\partial T}{\partial x_{1}}+{\overset{ˆ}{S}}_{12}^{-1}\frac{\partial T}{\partial x_{2}}+{\overset{ˆ}{S}}_{13}^{-1}\frac{\partial T}{\partial x_{3}}{|}^{\phi }\\ + & |{\overset{ˆ}{S}}_{21}^{-1}\frac{\partial T}{\partial x_{1}}+{\overset{ˆ}{S}}_{22}^{-1}\frac{\partial T}{\partial x_{2}}+{\overset{ˆ}{S}}_{23}^{-1}\frac{\partial T}{\partial x_{3}}{|}^{\phi }\\ + & {\left|{\overset{ˆ}{S}}_{31}^{-1}\frac{\partial T}{\partial x_{1}}+{\overset{ˆ}{S}}_{32}^{-1}\frac{\partial T}{\partial x_{2}}+{\overset{ˆ}{S}}_{33}^{-1}\frac{\partial T}{\partial x_{3}}{|}^{\phi }\right)}^{\frac{1}{\phi }}, \end{array}$$

where *ϕ* is the exponent describing the shape of the super-ellipsoid. Equation (12) has a simple solution for all ϕ∈N. The general form of the eikonal equation (13) is used later to compute the wave fronts in sandstone.

The procedure described in this section could also be reformulated to extract the metric tensor $g_{ij}$ on a Riemannian manifold. For that, the metric tensor $g_{ij}$ in the basis formed by normalizing the vectors in the slowness tensor is given by filling its diagonal with the slowness values in axes direction.

## 3 Results

Five experiments are presented in this section. If not mentioned explicitly, the experiments were executed using a grid of size 192×192×192 and a spacing of dx=dy=dz=0.7 meter. The first experiment shows the solution of the wave equation (9) for an isotropic velocity field. The solution was compared to an analytical solution of the eikonal equation to verify the validity of the proposed method. Next, a solution for a homogeneous velocity field with anisotropy is presented. This example can be motivated by the desire to approximate wave propagation through a homogeneously moving medium. The following example shows the result for an anisotropic and inhomogeneous velocity field as it could appear in simple real-life applications, motivated by an electric wave propagating through the heart muscles. The anisotropy is induced by the muscle fiber direction. For Experiment 4, we chose a velocity model that approaches real-life complexity as it comprises sharp velocity contrasts as found in many applications, especially in seismology. The last example shows the functionality of the method in media that shows a vertical transverse isotropy. This experiment is motivated by wave-motion modeling, executed in the scope of seismology.

For the first experiment, we are assuming the case of an isotropic homogeneous velocity field. The first velocity tensor is given at every point in the model space by

(14) $$\overset{ˆ}{V}=\overset{ˆ}{S}=\left(\begin{array}{ccc} 1 & 0 & 0\\ 0 & 1 & 0\\ 0 & 0 & 1 \end{array}\right).$$

**Figure 3:**
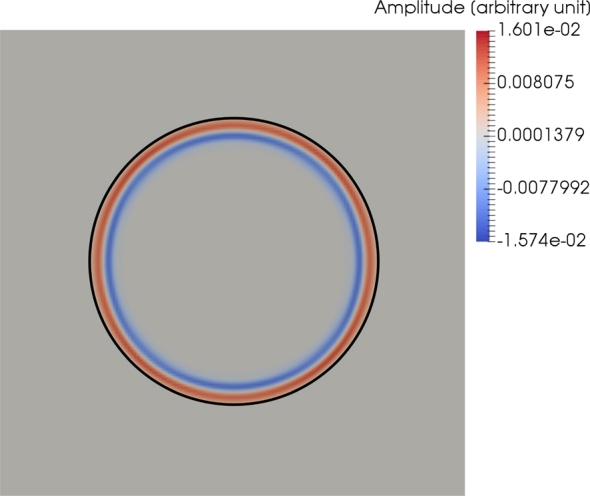
A two-dimensional slice of the three-dimensional wave field for the homogeneous velocity field. The wave front of the solution is compared to the analytical solution of the eikonal equation (black). The analytic solution of the isotropic eikonal equation aligns with the wave front of the solution of the wave equation (9).

Equation (14) represents the standard basis of the Euclidean space. Therefore, the velocity and slowness surfaces are spheres and the modeled velocity field is isotropic and homogeneous. Figure 3 shows the solution of the computation of equation (9). For proof of accuracy and correctness of equation (9), the analytic solution of the eikonal equation for isotropic media is included in Figure 3. For the given metric, the derived wave and eikonal equations could also be directly simplified to the equations for the isotropic and homogeneous case.

For Experiment 2, we are investigating a homogeneous, anisotropic velocity field. Now, the metric space is constant in the entire model and is given by the tensor

(15) $$\overset{ˆ}{V}=\left(\begin{array}{ccc} 1 & -2.0 & 0\\ 1 & 2.0 & 0\\ 0 & 0 & 1 \end{array}\right).$$

**Figure 4:**
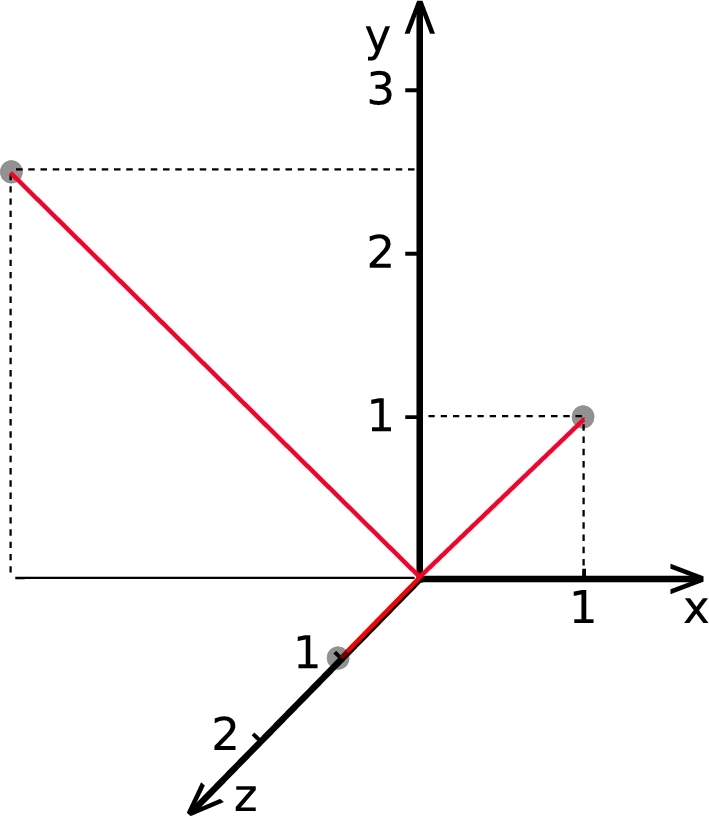
The basis of the new metric space (15) (in red) is shown with respect to the standard basis (black), for illustrative purposes. Note that the longest axis of the new metric is also the longest semi-principal axis of the ellipsoidal-shaped wave field shown in Figure 5.

For illustrative purposes, the basis is shown with respect to the standard basis in Figure 4. The corresponding velocity surface can be obtained by applying

(16) $${\overset{ˆ}{V}}^{-1}v=\left(\begin{array}{c} 0.5v_{1}+0.5v_{2}\\ -0.25v_{1}+0.25v_{2}\\ v_{3} \end{array}\right),$$

where the $v_{i}$s are the velocity components with respect to the original basis. Inserting the vector components of equation (16) in the velocity surface for isotropic wave propagation leads to

(17) $$1={\left(0.5v_{1}+0.5v_{2}\right)}^{2}+{\left(-0.25v_{1}+0.25v_{2}\right)}^{2}+v_{3}^{2}.$$

An approximation of the basis describing the slowness space can be obtained, as described in the theory section, by inverting the length of the basis vectors of the velocity tensor while maintaining their directions. The slowness surface can then be obtained by multiplying the inverse of the slowness tensor by the wave number **k**. For other forms of velocity surfaces the slowness surface is potentially much more difficult to find. This issue can be avoided by using the slowness surface in the solution process instead of the velocity surface. For this example, the velocity surface is chosen for illustrative reasons. However, the resulting slowness surface in this case is given by

(18) $$1={\left(p_{1}+p_{2}\right)}^{2}+{\left(-2p_{1}+2p_{2}\right)}^{2}+p_{3}^{2}.$$

**Figure 5:**
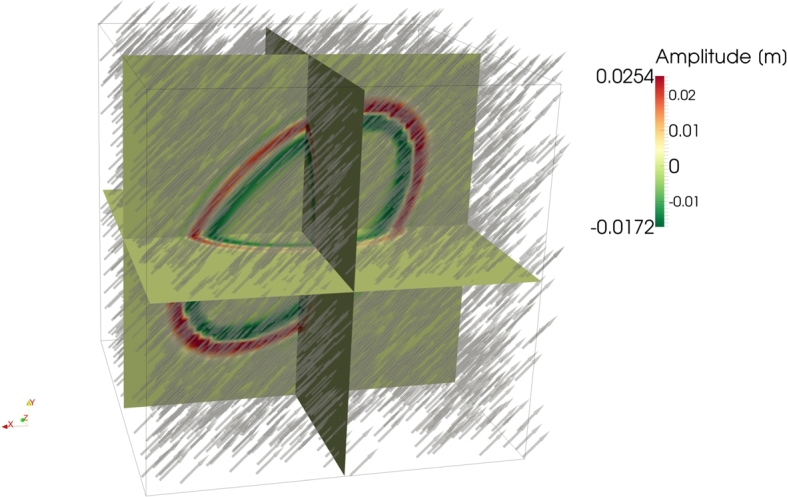
Three two-dimensional slices of the three-dimensional wave field of a homogeneous velocity field in the metric shown in Figure 4. The arrows show the longest axis ${\overset{ˆ}{V}}_{i2}$ of the velocity metric. The wave front represents a circle in the metric given by (15). Note, that the wave front resembles an ellipsoid in the Euclidean metric with the semi-principal axes given by the tensor (15).

In the case of ellipsoidal anisotropy, the required inverse of the slowness tensor is the transpose of the velocity tensor used to describe the basis for the velocity. This circumstance leads to faster computations since the creation of the slowness space and the inversion of the tensor can be omitted. After inserting $p_{i}=k_{i}/\omega$, multiplying by a function in the Fourier domain and an inverse Fourier transformation, as described in the theory section, equation (18) leads to the wave equation (9) for the given metric (15). A snapshot of the moving wave is shown in Figure 5. The results of this experiment could, for example, be applied to approximate wave propagation in a homogeneously moving medium.

**Figure 6:**
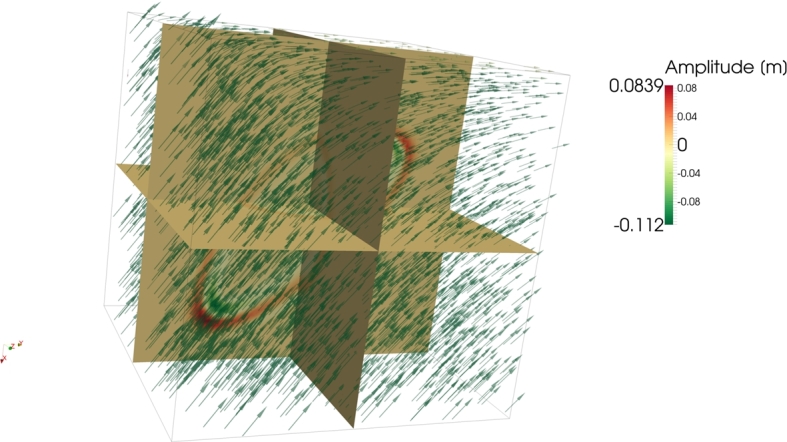
Three two-dimensional slices of the three-dimensional tensor field describing the velocity space and the corresponding wave field. The vectors are showing the longest axis ${\overset{ˆ}{V}}_{i1}$ of the given basis (19). ${\overset{ˆ}{V}}_{i2}$ and ${\overset{ˆ}{V}}_{i3}$ are perpendicular to the shown vector in each point and one third in length. Note, that the wave seems to follow a preferred direction given in each point in space by the tensor (19).

Wave propagation in inhomogeneous anisotropic media is the most important example for real-life applications and is investigated in Experiment 3. The given tensor depends on the position in the modeled space. The tensor field representing the basis and defining the metric, and therefore, the velocity anisotropy, is represented by its respective longest vector ${\overset{ˆ}{V}}_{i1}$ in Figure 6 together with the corresponding wave field. The velocity tensor is given by

(19) $$\overset{ˆ}{V}=\left(\begin{array}{ccc} \frac{-3x_{1}}{\sqrt{x_{1}^{2}+x_{2}^{2}}} & \frac{x_{2}}{\sqrt{x_{1}^{2}+x_{2}^{2}}} & 0\\ \frac{3x_{2}}{\sqrt{x_{1}^{2}+x_{2}^{2}}} & \frac{x_{1}}{\sqrt{x_{1}^{2}+x_{2}^{2}}} & 0\\ 0 & 0 & -1 \end{array}\right).$$

The axes ${\overset{ˆ}{V}}_{i2}$ and ${\overset{ˆ}{V}}_{i3}$ of the given basis are pairwise perpendicular to ${\overset{ˆ}{V}}_{i1}$, and one third of the length of ${\overset{ˆ}{V}}_{i1}$. A wave of this kind can be found in inhomogeneously moving media or in organs like the muscle tissue of the heart. In this case, the muscle fiber direction is responsible for the anisotropy.

**Figure 7:**
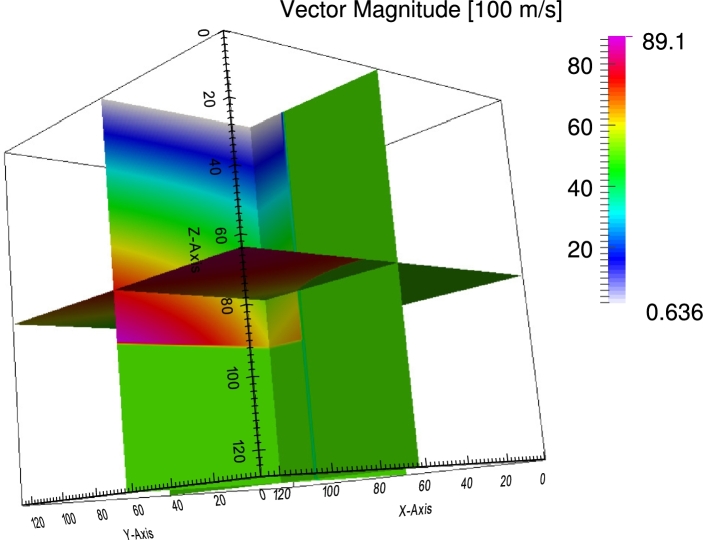
Velocity field comprising sharp velocity contrasts. The colors indicate the magnitude of the longest vector in the tensor describing the underlying metric space. Note, that the color, in this illustration, gives no information about the direction of the longest vector of the tensor. The directions are given in equation (20).

Most real-life applications of the proposed method will involve wave propagation through complex media. It is therefore important to test the method regarding its behavior when dealing with sharp velocity contrasts. One particular complex example of this kind is wave propagation through the geological subsurface and is the focus of Experiment 4. The velocity field in Figure 7 is defined on a grid of 128^3^ nodes and is given by

(20) $$\overset{ˆ}{V}=\left\{ \begin{array}{cc} \left(\begin{array}{ccc} 0 & 0 & 1\\ 0 & 20 & 0\\ 50 & 0 & 0 \end{array}\right) & \text{if}\sqrt{x_{1}^{2}+x_{2}^{2}}<80\\ \left(\begin{array}{ccc} \frac{-50x_{1}}{\sqrt{x_{1}^{2}+x_{2}^{2}}} & \frac{x_{2}}{\sqrt{x_{1}^{2}+x_{2}^{2}}} & 0\\ \frac{50x_{2}}{\sqrt{x_{1}^{2}+x_{2}^{2}}} & \frac{x_{1}}{\sqrt{x_{1}^{2}+x_{2}^{2}}} & 0\\ 0 & 100 & -1 \end{array}\right) & \text{if}\sqrt{x_{1}^{2}+x_{2}^{2}}<80\wedge x_{3}>80\\ \left(\begin{array}{ccc} \frac{x_{1}x_{3}}{\sqrt{x_{1}^{2}+x_{1}^{2}}} & \frac{x_{1}x_{2}}{\sqrt{x_{1}^{2}+x_{1}}} & 0\\ \frac{-x_{1}x_{3}}{\sqrt{x_{1}^{2}+x_{1}^{2}}} & \frac{x_{1}x_{2}}{\sqrt{x_{1}^{2}+x_{1}^{2}}} & \frac{1}{x_{1}}\\ \frac{x_{3}}{\sqrt{x_{1}^{2}+x_{2}^{2}}} & 0 & 30 \end{array}\right) & \text{else}. \end{array}\right.$$

**Figure 8:**
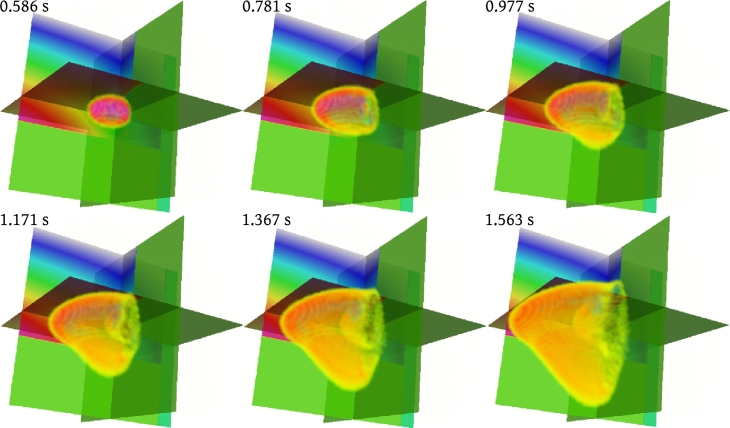
Snapshot of the wave field after indicated times. Note the behavior of the solution at interfaces between materials with different preferred directions of wave propagation. Note, that no artifacts emerge in the solution.

The velocity model in Figure 7 comprises two layers with different preferred propagation directions and a body whose preferred propagation direction is perpendicular to the ones of the two layers. The model contains sharp interfaces and strong velocity variations. Snapshots of the three-dimensional wave field are shown in Figure 8.

**Figure 9:**
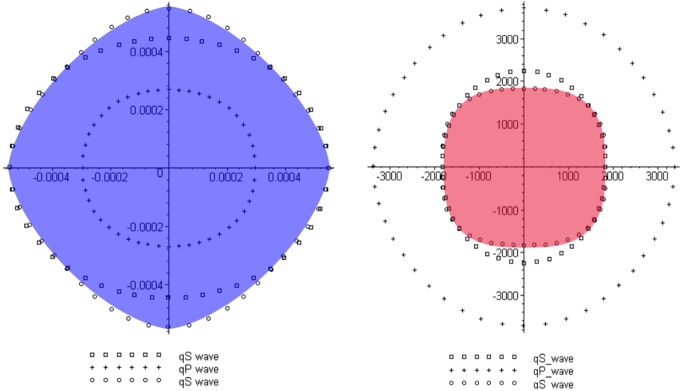
The velocity and slowness surfaces of different wave modes in sandstone. The super-ellipsoid $x_{1}^{\frac{3}{2}}+x_{2}^{\frac{3}{2}}+x_{3}^{\frac{3}{2}}=1$ (in blue) corresponds to the s-wave slowness surface. The solution resembles the shape marked in red. Figure modified from Piedrahita et al. [36].

Slowness or velocity surfaces in real materials are often not elliptical. To verify the functionality of the method for wave propagation in materials showing other velocity-surface shapes, Experiment 5 presents a result for sandstone. Sandstone is typically considered to have vertical transverse isotropy (VTI). This name describes a medium whose parameters are invariant regarding a rotation around the z-axis [36]. As represented in Figure 9, the super-ellipsoid $|x_{1}{|}^{\frac{3}{2}}+|x_{2}{|}^{\frac{3}{2}}+|x_{3}{|}^{\frac{3}{2}}=1$ describes the velocity surface of the s-wave with reasonable accuracy considering a certain simplicity which shall be maintained. From Figure 9, the following slowness surface can be derived

(21) $$\omega^{\frac{3}{2}}=k_{1}^{\frac{3}{2}}+k_{2}^{\frac{3}{2}}+k_{3}^{\frac{3}{2}}.$$

Using the proposed method, the following eikonal equation can be derived

(22) $$\begin{array}{cc} 1=( & |{\overset{ˆ}{S}}_{11}^{-1}\frac{\partial T}{\partial x_{1}}+{\overset{ˆ}{S}}_{12}^{-1}\frac{\partial T}{\partial x_{2}}+{\overset{ˆ}{S}}_{13}^{-1}\frac{\partial T}{\partial x_{3}}{|}^{\frac{3}{2}}\\ + & |{\overset{ˆ}{S}}_{21}^{-1}\frac{\partial T}{\partial x_{1}}+{\overset{ˆ}{S}}_{22}^{-1}\frac{\partial T}{\partial x_{2}}+{\overset{ˆ}{S}}_{23}^{-1}\frac{\partial T}{\partial x_{3}}{|}^{\frac{3}{2}}\\ + & {\left|{\overset{ˆ}{S}}_{31}^{-1}\frac{\partial T}{\partial x_{1}}+{\overset{ˆ}{S}}_{32}^{-1}\frac{\partial T}{\partial x_{2}}+{\overset{ˆ}{S}}_{33}^{-1}\frac{\partial T}{\partial x_{3}}{|}^{\frac{3}{2}}\right)}^{\frac{2}{3}}. \end{array}$$

**Figure 10:**
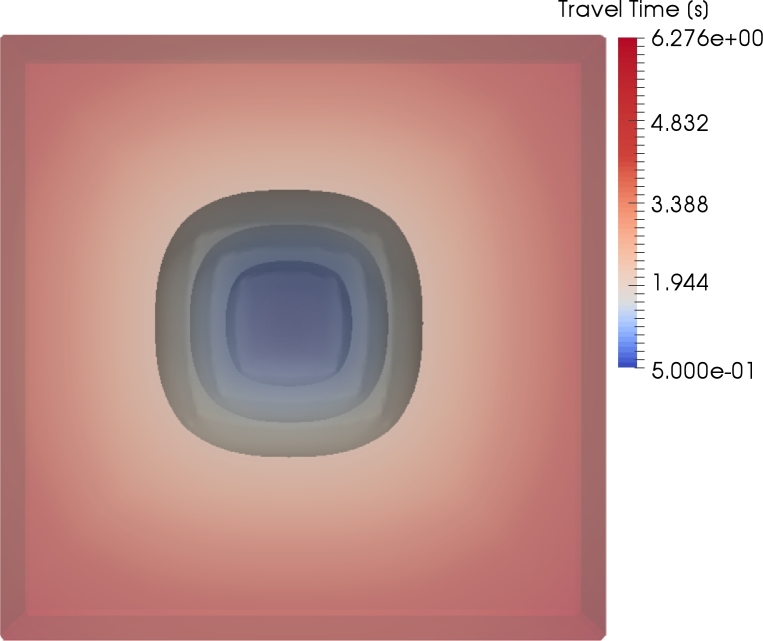
The solution of equation (22). Note that there is a high level of resemblance between the solution and the shape shown in Figure 9.

The solution of equation (22) is presented in Figure 10. For simplicity, the slowness tensor is spatially independent and not tilted. The eikonal equation (22) and the associated wave equation are valid for tilted and inhomogeneous sandstone.

## 4 Discussion

The results showed the accuracy and the functionality of the method for anisotropic, homogeneous and inhomogeneous velocity fields. The comparison with an analytical solution of the eikonal equation proved that the wave front of the solution resembled the analytical wave front. Figure 3 showed that the analytical solution of the eikonal equation aligns with the solution of the derived wave equation (9).

The result of the second experiment (Figure 5) showed the solution of equation (9) for a homogeneous anisotropic velocity field, approximating, for example, a moving medium. The wave front of the resulting wave has the expected shape of an ellipsoid given by a transformed sphere into the new metric space (15).

The result of the third experiment (Figure 6) showed the solution of equation (9) for an inhomogeneous anisotropic velocity. Such a velocity field can occur in nature, for instance, in muscle tissue or in inhomogeneously moving media. The resulting wave field showed that the wave follows the preferred propagation direction given in every point in space by the tensor (19).

Experiment 4 tested the method regarding the wave propagation through a synthetic, geological subsurface. It turned out, that the method handles sharp velocity contrasts in a stable manner. No artifacts appear in the solution as in Alkhalifah [18], [37]. This is due to the fact that, from a physical perspective, the method propagates acoustic waves in a homogeneous, isotropic medium; what changes is the underlying space. The last experiment showed the solution of an eikonal equation derived by the proposed method of a s-wave propagating through sandstone. In this case, sandstone exhibits vertical transverse isotropy. The resulting wave front (see Figure 10) resembles the expected wave front depicted in Figure 9. Again, artifacts do not appear in the solution as in Alkhalifah [37]. This result demonstrates the ability of the method to be applied to other slowness surfaces and therefore types of anisotropy apart from elliptical ones. However, the equations can become complex for non-integer exponents in the dispersion relation.

The problem of transforming the velocity surface into a slowness surface is not method-specific and can be avoided by dealing with slowness surfaces in the solution process in the first place, or by acknowledging that the accurate velocity and slowness surfaces are unknown in practice. The proposed derivation method for acoustic wave propagation problems offers a straight-forward derivation and implementation. In cases where only travel times are important, the proposed method can be used to derive eikonal equations for various types of anisotropy. The proposed method can also be used to derive wave equations for any kind of velocity or slowness surface. Here, velocity surfaces in the form of a super-ellipsoid were discussed for simplicity. The corresponding wave and eikonal equations are simple to implement and the computation proved to be stable. The solution does not show any artifacts. The method therefore, has a large potential benefit for research and industry fields in which wave propagation in anisotropic media plays a significant role. Especially the fields of bio-computing, and seismology could benefit from the method. Wave propagation through metamaterials can be described and modeled in an efficient and simple manner. Also, acoustic wave propagation through moving media like waves traveling through water or air can be approximated in a straight-forward way.

We are claiming that the proposed method leads to a simpler derivation of the governing equations, a simple implementation and an efficient and stable computation. These assertions will be challenged in this paragraph. The simple derivation mainly stems from the fact, that we are interpreting changes of material parameters in certain directions as a change of the underlying metric space. The benefits are two fold. Firstly, we are only dealing with velocity surfaces instead of elastic parameters, which is very comprehensible. Secondly, we can use a clean description of velocity by using bases and norms, which allows for a simple derivation of the governing equations. A drawback is that we have to calculate a tensor representation of the velocity, in case we are dealing with elastic parameters only. The methods works best if the starting points are velocity surfaces. In this case, we can challenge the simplicity statement. We can compare the derivation of the eikonal equation by Alkhalifah [18] to the derivation of the eikonal equation (22) for sandstone in this paper. Equation (22) has a simpler structure. However, the complexity of an equation remains subject to personal preference. Another great example for the computation of wave phenomena in anisotropic media is the work of Joets and Ribotta [38]. The computation of the eikonal equation obtained by using the proposed method includes the computation of all ray directions and has a simpler form. However, the method described by Joets and Ribotta [38] is more general. An advantage of the proposed method is, that the equations will only change slightly for different kinds of anisotropy within certain limits, which are discussed in more depth later on. Again, if the starting point are elastic parameters, the derivation by Alkhalifah [18] is about as simple as the proposed approach. For a further evaluation of simplicity, we can have a look at Cervený et al. [35]. The eikonal equation for anisotropy is derived by using the eigenvalues of the Christoffel matrix, which is not a simple concept compared with dealing with changes in the metric and basis transformation. The simple implementation comes from the fact that the algorithm is basically a solver of the acoustic, isotropic wave or eikonal equation in homogeneous media. The only additional work goes into changing exponents and inverting simple matrices. The close relation to an acoustic solver for homogeneous, isotropic media is also the reason for the stability and efficiency of the computation. Here, we have to address another limitation. The proposed method can lead to fractal derivatives in the wave equation which can compromise the computational efficiency. The inclusion of boundary conditions, using the proposed approach, is simple and follows the procedure for the derivation of wave equations. The computational efficiency stems mainly from the fact that the additional computations, namely an inversion of a 3 matrix can be done efficiently on GPU cores, which is the preferred architecture for wave-motion simulations. Therefore, the computational efficiency of the solution of the eikonal equation does not depend on the kind of anisotropy within our defined set of anisotropies. In general, it can be said, that the method's strength is repeatability of derivations and implementations.

The limitations of the method can be clearly formulated. The method is, by construction an approximation. However, depending on the allowed complexity, this approximation can induce smaller errors than, for example, the approximation of the real medium as a model given some information. Also, the method breaks down as soon as triplications occur in the slowness surface. In this work, the slowness surface had to be in the form of a super-ellipsoid. However, this is just a limitation of derivation, not a basis limitation of the method, since it is, in theory, possible to extend the derivation to more general surfaces.

In this paper, the focus was on super-ellipsoidal surfaces and, in particular, ellipsoidal and vertical transverse isotropy, which was approximated by the proposed method. In future work, other kinds of surfaces could be investigated with respect to complexity and computational feasibility. Special interest lies on velocity surfaces described by super-ellipsoids with exponents that are not elements of the natural numbers and higher order surfaces. Also, more general shapes, like spherical harmonics, could be used to derive wave equations for complex types of anisotropy. The proposed theory could, because of its simplicity of derivation and application, build a new basis for the investigation of acoustic wave propagation in anisotropic media.

## 5 Conclusion

A new method for deriving wave and eikonal equations for acoustic wave propagation in anisotropic media was presented and validated by experiments. The proposed theory generalizes various types of anisotropy by narrowing the procedure down to the selection of a slowness or velocity surface, and a tensor field defining a new metric space at each spatial model point, thereby simplifying the derivation of the governing equation. Since all the changes are with regard to the underlying space, the numerical computations are as stable as the computations in isotropic media. No artifacts can be seen in the solutions as in Alkhalifah [18], [37]. In this work, we covered surfaces which can be described as a super-ellipsoid. A greater variety of slowness surfaces will be addressed in future work.

## Declarations

### Author contribution statement

Marcus M. Noack: Conceived and designed the experiments; Performed the experiments; Analyzed and interpreted the data; Contributed reagents, materials, analysis tools or data; Wrote the paper.

Stuart Clark: Analyzed and interpreted the data; Contributed reagents, materials, analysis tools or data; Wrote the paper.

### Funding statement

This work was supported by Kalkulo AS and the Research Council of Norway (grant 238346).

### Competing interest statement

The authors declare no conflict of interest.

### Additional information

No additional information is available for this paper.
